# Targeted Deletion of a *Plasmodium* Site-2 Protease Impairs Life Cycle Progression in the Mammalian Host

**DOI:** 10.1371/journal.pone.0170260

**Published:** 2017-01-20

**Authors:** Konstantinos Koussis, Evi Goulielmaki, Anna Chalari, Chrislaine Withers-Martinez, Inga Siden-Kiamos, Kai Matuschewski, Thanasis G. Loukeris

**Affiliations:** 1 Institute of Molecular Biology and Biotechnology, Foundation for Research and Technology-Hellas, Heraklion, Greece; 2 Parasitology Unit, Max Planck Institute for Infection Biology, Berlin, Germany; 3 Malaria Biochemistry Laboratory, The Francis Crick Institute, London, United Kingdom; 4 Department of Biology, University of Crete, Heraklion, Greece; 5 Institute of Biology, Humboldt University, Berlin, Germany; Université Pierre et Marie Curie, FRANCE

## Abstract

Site-2 proteases (*S2P*) belong to the M50 family of metalloproteases, which typically perform essential roles by mediating activation of membrane–bound transcription factors through regulated intramembrane proteolysis (RIP). Protease-dependent liberation of dormant transcription factors triggers diverse cellular responses, such as sterol regulation, Notch signalling and the unfolded protein response. *Plasmodium* parasites rely on regulated proteolysis for controlling essential pathways throughout the life cycle. In this study we examine the *Plasmodium*-encoded *S2P* in a murine malaria model and show that it is expressed in all stages of *Plasmodium* development. Localisation studies by endogenous gene tagging revealed that in all invasive stages the protein is in close proximity to the nucleus. Ablation of *PbS2P* by reverse genetics leads to reduced growth rates during liver and blood infection and, hence, virulence attenuation. Strikingly, absence of *PbS2P* was compatible with parasite life cycle progression in the mosquito and mammalian hosts under physiological conditions, suggesting redundant or dispensable roles *in vivo*.

## Introduction

Regulated Intramembrane Proteolysis (RIP) is a widely conserved signalling transduction mechanism, which involves the proteolytic processing and release of cytoplasmic or extracellular proteins from transmembrane precursors. These transmembrane cleavage events are mediated by four families of polytopic intramembrane cleaving proteases (i-CLiPs), which include the Rhomboid family of serine proteases, the γ-secretase and Signal Peptide Peptidase families of aspartic proteases, and the Site-2 Protease family of metalloproteases.

**Fig 1:**
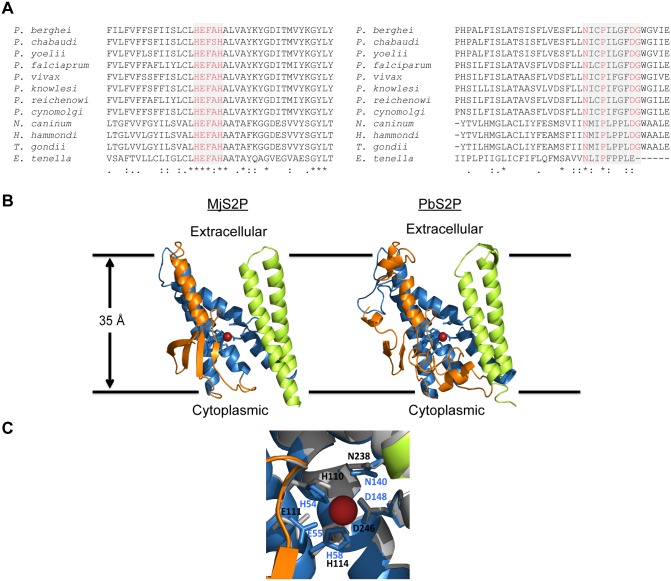
*Plasmodium* M50 proteases. (A) Conserved catalytic motifs (HExxH and NxxPxxxxDG- highlighted red in grey boxes) from a multiple sequence alignment of S2P orthologues from *Plasmodium* species and related apicomplexan parasites. (B) 3D homology model of *Pb*S2P (right panel—PbANKA_1404100) using the open conformation of *Methanocaldococcus jannaschii* S2P (left panel—PDB id: 3B4R) as a template and Phyre2 as program. The first transmembrane domain is labelled in orange, the second to fourth in blue, and the fifth and sixth in lime green, respectively. The catalytic zinc atom is depicted in red and the catalytic residues are shown surrounding the zinc atom as blue sticks. The orientation within the lipid membrane is also indicated. (C) Magnification of the active site in the *Pb*S2P homology model, illustrating the structural conservation of the catalytic residues. Strictly conserved residues are shown as sticks and are labelled in black for *Pb*S2P and blue for the respective homologous amino acid residues in *M*. *jannaschii* S2P.

The human Site-2 Protease (S2P) was the first i-CLiP to be identified almost two decades ago as one of the processing proteases for the sterol regulatory element binding proteins (SREBPs) [1]. Subsequent studies identified similar mechanisms in bacteria and plants. Today it is known that S2Ps are present in all kingdoms of life, apart from viruses and certain bacteria species with small size genomes such as *Mycoplasma* [2]. Together, they form a distinct family of polytopic membrane metalloproteases, termed M50, with members containing 4 to 8 transmembrane domains. All members of the family have a conserved 3 transmembrane domain core structure containing the metalloprotease characteristic HExxH motif within the first transmembrane domain of the core. A second highly conserved motif, Asn-(x)_2_-Pro-(x)_4_-Asp-Gly (abbreviated NPDG), resides in the third transmembrane domain of the core structure (Fig 1A and 1B). The active site is formed by the Asp residue in the NPDG motif and the two His residues of the HExxH motif [2, 3].

Members of the M50 family perform diverse, and typically essential, proteolytic functions. In prokaryotic organisms, for instance, S2Ps are critical for environmental stress responses, sporulation, cell division, pheromone production, mucoid production and iron uptake [4]. The best characterised S2P regulated signalling pathway in bacteria is the *E*.*coli* sigma factor E (SigE) stress response, controlled by the RseP homolog of S2P [5]. S2P-mediated signalling pathways are also utilised by many bacterial pathogens. Prominent examples include the *Vibrio cholerae* YaeL, which regulates degradation of a membrane-associated virulence activator and thereby shuts down virulence related genes under non-favourable growth conditions [6], and Rip1 in *Mycobacterium tuberculosis*, a critical virulence regulator involved in the proteolysis of four sigma factors [7, 8].

In mammals, S2P is a Golgi-resident protease, participating in two physiological pathways involving lipid metabolism and the unfolded protein response (UPR) [1, 9]. When cholesterol levels drop, a series of regulated events is activated, leading to the translocation of SREBP transcription factor, which harbours two transmembrane spans, from the ER to the Golgi. Next, SREBP undergoes a two-step proteolytic processing, first by a serine protease termed Site-1 protease (S1P) within the luminal loop of the two transmembrane domains, followed by an intra-membrane cleavage of the amino-terminus, which contains the transcription factor, by S2P. Translocation of active SREBP to the nucleus activates genes involved in the production and trafficking of cholesterol, fatty acids and other lipids [1, 10, 11]. A similar cascade of events, involving the Activating transcription factor 6 (ATF6), occurs when the UPR is activated. ATF6 is transported from the ER to the Golgi, where it is sequentially cleaved by the S1P/S2P protease pair. The latter proteolytic event releases the cytosolic DNA-binding portion, which is transported to the nucleus in order to initiate transcription of ER chaperone genes [9, 12]. Further studies have shown that homologues of ATF6, such as CREBH in the liver and OASIS in astrocytes, are also regulated by RIP mediated by the S1P/S2P pair [13, 14].

*Plasmodium* parasites, the causative agent of malaria, are single cell eukaryotes that rely on proteases to control essential biological functions ranging from parasite development and haemoglobin degradation to invasion and egress [15, 16]. *Plasmodium* genomes encode three i-CLiP families, while γ-secretases have not yet been identified. The Rhomboid family of i-CLiPs in *Plasmodium* and related apicomplexan parasites has been extensively studied, because of their importance in host cell invasion and egress [17]. *Plasmodium* parasites encode eight rhomboids. ROM1 and ROM4 are known *in vitro* to cleave different adhesins involved in cell invasion and attachment but for the other rhomboids no substrates have been identified [18–20]. A systematic reverse genetics analysis in the mouse malaria model *Plasmodium berghei* has shown that four genes, *ROM4*, *ROM6*, *ROM7* and *ROM8*, are refractory to targeted gene deletion in asexual blood stages, while *ROM3* is essential for sporozoite production in the *Anopheles* vector and the remaining three, *ROM1*, *ROM9* and *ROM10*, are dispensable for life cycle progression [21]. The *P*. *falciparum* signal peptide peptidase is also refractory to gene deletion suggesting an essential role in the intra-erythrocytic development of the parasite and the data available are consistent with an important role in ER homeostasis [22–24].

In most *Apicomplexa*, genes encoding M50 proteases have been identified [25], but to date no experimental studies are available. In this work, we characterize the *P*. *berghei* S2P protease (PBANKA_1404100/*Pb*S2P). Gene ablation is compatible with parasite replication and stage conversion but results in reduced parasite development in late liver stages and in the asexual intra-erythrocytic cycle.

## Materials and Methods

### Ethics statement

All animal work has passed an ethical review process and was approved by the FORTH Ethics Committee (FEC). All work was carried out in accordance to the Greek regulations: Presidential Decree (160/91) and law (2015/92) which implement the directive 86/609/EEC from the European Union and the European Convention for the protection of vertebrate animals used for experimental and other scientific purposes and the new legislation Presidential Decree 56/2013. The experiments were carried out in a certified animal facility (license number EL91-BIOexp-02) and with a project license (#27290 to ISK), and some of the animal work was conducted in accordance with approval by Berlin state authorities (LAGeSo Reg# G0469/09 and G0294/15). Animals were kept in dedicated facilities. Air is filtered and recycled 12–15 times/hour *via* HEPA filters. Temperature is controlled at ~24°C and humidity 55+10%, and light is on a day-night cycle. Temperature and humidity are recorded continually. Mice are kept in cages with a minimal height 120 mm and 80cm2 floor area/animal and minimum area 330 cm2. Cages are covered by steel grid, while food and water is provided *ad libidum*. Animals were monitored daily and for ECM experiments twice daily at 9h00 and 18h00 with no unexpected deaths occurred during these experiments. Humane endpoints were used during this study based on levels of parasitaemia as determined by Giemsa-stained blood smears and on initial development of ECM symptoms. Animals reaching these points were euthanized by cervical dislocation under deep anaesthesia. All experiments are considered causing mild distress and all invasive procedures were carried out under anaesthetics (ketamine) and animals were sacrificed immediately after the procedure was completed.

### Parasite maintenance

*P*. *berghei* line 507 expressing GFP constitutively [26] and *P*. *berghei* ANKA were maintained in 6–10 week-old Theiler’s Original (OlaTO) and NMRI mice. C57BL/6 mice were used for sporozoite infections and parasite virulence studies and BALB/c mice for cloning. *Anopheles stephensi* or *Anopheles gambiae* G3 strain were used in all mosquito infections following standard procedures.

### DNA constructs

The *PbS2P* knockout construct was made in the standard vector pL0001 (www.mr4.org). 495 bp of the 5′ region of *PbS2P* ORF and a 494 bp fragment of the 3′ end of the *PbS2P* ORF were amplified from *P*. *berghei* genomic DNA (gDNA) using primers KD5For/KD5Rev and KD3For/KD3Rev, respectively. Primers used in this study are shown in S1 Table. Fragments were cloned *via Kpn*I/*Hind*III and *EcoR*V/*BamH*I, respectively. Prior to transfection the plasmid was linearized with *Kpn*I and *Xba*I. For tagging of *Pb*S2P with a 3xHA tag, vector pBGEM-062856 from the PlasmoGEM resource was used [27, 28]. This vector was digested with *NotI* prior to transfection.

### Transfection, cloning and genotyping

Schizonts for transfection were purified from overnight infected red blood cell cultures and transfected with 10 μg of linearised DNA as previously described [26]. Resistant parasites were selected by pyrimethamine (70 mg/l) supplied in the drinking water. All transgenic parasites were cloned by limiting dilution and clonal lines were verified by diagnostic PCR and/or Southern blot. Southern blot analysis was done using North2South^™^ Chemiluminescent Hybridization and Detection Kit (ThermoFisherScientific) following manufacturer’s instructions.

### Intraerythrocytic and mosquito stages phenotypic analysis

Intraerythrocytic growth was measured by intravenous injection of 10,000 or 1,000 infected erythrocytes into naïve recipient C57BL/6 mice. Parasitaemia was determined by Giemsa-stained blood films. Exflagellation was measured 4 days post infection of phenylhydrazine-treated OlaTO mice with 1x10^6^ infected red blood cells. 2 μl of infected blood were incubated for 10 min at 19°C in 10 μl of complete ookinete culture medium (RPMI1640 containing 25 mM HEPES (Sigma), 10% FCS, 50 μM xanthurenic acid, pH 7.5). Formation of exflagellation centres was measured by phase contrast microscopy counting 10–12 fields. *P*. *berghei in vitro* ookinete culture and purification was performed as previously described [29]. Ookinete conversion rates were calculated using a mouse monoclonal antibody (13.1), which recognizes the P28 protein [30]. For the standard membrane feeding assays, enriched ookinetes were resuspended in fresh blood from naive mice at a density of 3-5x10^3^ ookinetes/μL and placed into water jacketed glass feeders (37°C), at a volume of 0.1–0.2 ml/feeder. The mixture was offered to 5–7 day old female *A*. *gambiae* mosquitoes, after which unfed females were removed. Midguts were dissected and examined for the presence of oocysts 10 days after feeding.

### Analysis of *in vivo* sporozoite infectivity

Salivary gland sporozoites were isolated and numbers were determined at days 19–21 after feeding of *A*. *stephensi* mosquitoes with WT or *s2p(-)*-infected mice. To determine sporozoite infectivity, C57BL/6 mice were challenged by natural bite with 6–8 infected mosquitoes or intravenous injection of 10,000 sporozoites. Patency was determined by microscopic examination of Giemsa-stained blood films starting at day 3 after infection. During the analysis C57BL/6 mice were monitored for the development of behavioural and functional symptoms associated to ECM [31]. Immediately after diagnosis of ECM, mice were sacrificed.

To measure parasite load in the liver, 10,000 sporozoites were injected intravenously into C57BL/6 naïve mice. 42 hours after infection, livers were harvested for RNA extraction and cDNA synthesis using the Tri Reagent (Sigma) and RETROScript kit for cDNA (Ambion). Gene-specific primers for *P*. *berghei* 18S rRNA and the mouse *GAPDH* gene were used for amplification (S1 Table). Relative transcript abundance was determined as previously described [32]. Real time PCR was performed on the StepOnePlus^™^ Real-Time PCR System using the Power SYBR^®^ Green PCR Master Mix (Applied Biosystems), following manufacturer’s instructions.

### Analysis of *in vitro* sporozoite development

Liver stages were cultured *in vitro* and analyzed as described [33]. Briefly, 7,500 hepatoma (Huh7) cells were seeded in wells of a 96 well plate and infected 24h later with 10^4^ salivary gland sporozoites. Cells were fixed, at 24 and 48h after infection with 4% paraformaldehyde. Staining of exoerythrocytic forms (EEFs) was done with a mouse α-*Pb*HSP70 Ab (dilution 1:300) [34]. For enumeration of merosomes, infected Huh7 were cultured for up to 72 h and merosomes were collected from cell culture supernatants.

### Real time quantitative RT-PCR

Parasite total RNA was extracted with Tri Reagent (Sigma) and cDNA was made using the RETROScript kit (Ambion). Real time PCR was performed as above. Expression data were normalized to the constitutively expressed *GFP* and *PbHSP70* genes.

### Indirect immunofluorescence analysis (IFA) and immunoblot analysis

Schizonts were smeared onto glass slides, air-dried, fixed in 4% formaldehyde (in PBS) for 20 min, permeabilised in 0.1% (v/v) Triton X-100 and blocked for 2h in 3% (w/v) bovine serum albumin (BSA) in PBS. Slides were probed overnight at 4°C with α-HA (3F10, Roche; 1:100 dilution) and mouse α-HSP70. For the colocalisation studies, a rabbit α-ERD2 antibody was used at 1:1,000 dilution (kind gift of Prof. David Baker, LSHTM-London). Enriched ookinetes were allowed to settle on coverslips pre-treated with poly-L lysine for 30 minutes. Ookinetes were treated as described above using a mouse α-MTIP (1:500 dilution) for double labelling. The antiserum directed against MTIP was obtained after injection of a plasmid construct with the complete ORF of MTIP cloned in the pSecTag2 vector (Thermo Fisher Scientific) in BALB/C mice. Salivary gland sporozoites were fixed on glass slides for 1h in 4% formaldehyde/0.0075% glutaraldehyde, permeabilised and blocked as described above. Primary antibodies used were the α-HA (1:100) and α-MTIP (1:500). All antibodies were diluted in 1% BSA/PBS/0.1% Triton X-100. For oocyst IFA, infected mosquito midguts were dissected and fixed in 4% formaldehyde, 0.2% saponin (Sigma) in PBS for 45–60 min. Washes and antibody dilutions were done in PBS with 0.2% saponin and 3% BSA. After addition of α-HA (1:00) and α-*Pb*Cap380 (1:1,000) [35], the midguts were kept at 4°C overnight. Mosquito midgut epithelial sheets were prepared for IFA as described elsewhere [36, 37]. Samples were incubated with α-SRPN6 [37], α-P28 monoclonal antibody (1:1,000) and α-PbCap380 (1:1,000).

In all IFAs, secondary antibodies were added for 1 h (diluted 1:1,000) and cells were mounted in Vectashield^™^. The following secondary antibodies were used: Alexa Fluor 488-labelled goat anti-rabbit IgG, Alexa Fluor 488-labelled goat anti-mouse IgG and Alexa Fluor 555 or 488 labelled goat anti-rat (all from Molecular probes). Nuclei were stained with TO-PRO 3 (Molecular probes, diluted 1:1,000) or Hoechst-33342 (Invitrogen, diluted 1:5,000). Images were collected using a Leica TCS SP2 confocal laser scanning microscope with Biorad lasers or a Zeiss Axioskop 2 plus microscope fitted with an Axiovert CCD camera (Zeiss). Images were analysed with Fiji software.

For Western-blot analysis, 10^6^ cells (schizonts) were lysed in RIPA buffer (50 mM Tris-Cl pH 8.0, 150 mM NaCl, 2 mM EDTA, 1% NP-40, 1% Sodium Deoxycholate, 0.1% SDS) supplemented with Protease Inhibitor Cocktail (Sigma). Samples were processed for Western blot analysis as has been previously described [38] using rat α-HA antibody (3F10, Roche; 1:1,000) and goat anti-rat HRP conjugate as a secondary antibody (Invitrogen; 1:10,000). The blot was developed using the ECL system (SuperSignalWest Pico, Thermo Scientific) following manufacturer’s instructions.

### Bioinformatic analysis, structural modelling and model evaluation

Multiple alignments were performed using T-Coffee from the EMBL_EBI server and editing with ESPript 3.0 [39]. PBANKA_1404100 structural modelling was carried out using the Phyre2 web portal for protein modelling, prediction and analysis [40]. The program highest confidence template, the zinc site-2 protease from *Methanocaldococcus jannaschii* (PDB id: 3B4R, 100% probability for homology, 19% identity) was selected for modelling PBANKA_1404100. The resulting model was analysed within the PyMOL Molecular Graphics System, Version 1.8 Schrödinger, LLC, (http://www.pymol.org). Images were also produced in PyMOL.

### Statistical analysis

Statistical significance was assessed by standard statistical methods, with a *P* value of <0.05 considered as a significant difference. All of the statistical tests were performed with GraphPad Prism 6 (GraphPad Software).

## Results

### *Plasmodium* genome encodes one M50 protease

*Plasmodium berghei* encodes a single member of the M50 family of proteases (PBANKA_1404100), henceforth termed *PbS2P*. The protein is predicted to have 7 transmembrane domains and a molecular weight of 39 kDa. *In silico* searches in PlasmoDB (http://plasmoDB.org) and EupathDB (http://eupathdb.org) identified genes encoding M50 metalloproteases in all *Plasmodium* species and in most organisms of the *Apicomplexa* phylum, with the notable exceptions of *Babesia* and *Cryptosporidium* species. All proteins contain the characteristic HExxH motif and only one (*Eimeria tenella*) does not contain the NPDG motif (Fig 1A and S1 Fig). *Pb*S2P has an overall amino acid identity of more than 92% with other rodent malarial parasite S2Ps; a value that drops to 70% identity when compared to human parasite orthologues and to less than 30% in S2Ps of related *Apicomplexa*, such as *Toxoplasma* and *Neospora*, and other eukaryotic organisms or bacteria (S1 Fig).

Homology modelling of *Pb*S2P was based on the only available S2P crystal structure available from *Methanocaldococcus jannaschii* (PDB id: 3B4R) [41]. The overall root-mean-square deviation (RMSD) of the backbone coordinates between the two molecules is 0.90 Å, with even lesser deviation in the vicinity of the active site. *Pb*S2P is predicted to also have seven transmembrane regions but fewer β-strand elements when compared to the template (Fig 1B). In the active site, the three S2P invariant amino acids coordinating the catalytic zinc atom are structurally conserved with H110 and H114 from helix α2 and D246 from α4. There is also structural conservation of residue E111, suggested to activate a zinc-bound water molecule for catalysis initiation. Finally, the putative oxyanion hole partner N238 is also structurally conserved (Fig 1C). Overall, the degree of amino acid conservation in the vicinity of the active site together with the striking structural homology of the two molecules strongly indicates that this molecule belongs to the S2P protease family.

### *Pb*S2P is expressed throughout the life cycle and localises in proximity to the nucleus

**Fig 2:**
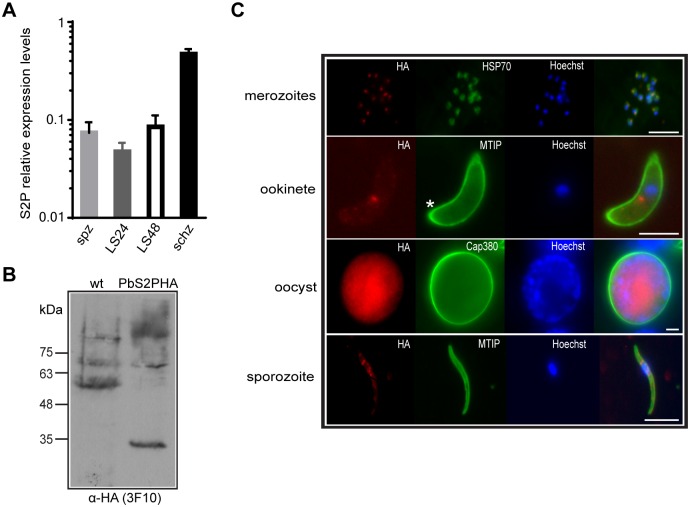
Expression and localisation of *Pb*S2P. (A) Relative expression levels of *PbS2P* as determined by qRT-PCR from cDNAs of schizonts (schz), sporozoites (spz), 24h liver stages (LS24) and 48h liver stages (LS48). Transcript levels were normalised to *PbHSP70* and *GFP*. (B) Western blot analysis of *Pb*S2P-HA whole protein extract from purified schizonts of transgenic *PbS2P-HA* parasites using an α-HA antibody. *Pb*S2P-HA migrates at 35kDa. (C) Immunofluorescence analysis (IFA) of *Pb*S2P-HA merozoites, ookinete, oocyst, and salivary gland sporozoites using α-HA (3F10) for detection of *Pb*S2P (red) and Hoechst stain for the nucleus (blue). For delineation of parasites the following antibodies (green) were used: α-HSP70, schizonts/merozoites; α-MTIP, ookinete and sporozoite; α-PbCap380, oocyst. Prominent localisation of *Pb*S2P in proximity to the nucleus is present in all invasive stages. Star, apical end of ookinete. Scale bar 5 μM.

RNA-seq data on gene expression of rodent malaria species [42] has shown that *PbS2P* is expressed in asexual blood stages, gametocytes and early mosquito stages of zygote/ookinetes with the highest expression levels at the schizont stage. To further characterise *PbS2P*, we expanded transcript profiling to salivary gland sporozoites and liver stages grown in hepatoma cells. cDNA samples were prepared and relative expression levels were measured by RT-qPCR alongside a schizont cDNA sample. Transcripts of *PbS2P* were detected, at reduced levels in salivary gland sporozoites, and at 24h and 48h after hepatocyte invasion compared to synchronized schizonts (Fig 2A).

**Fig 3:**
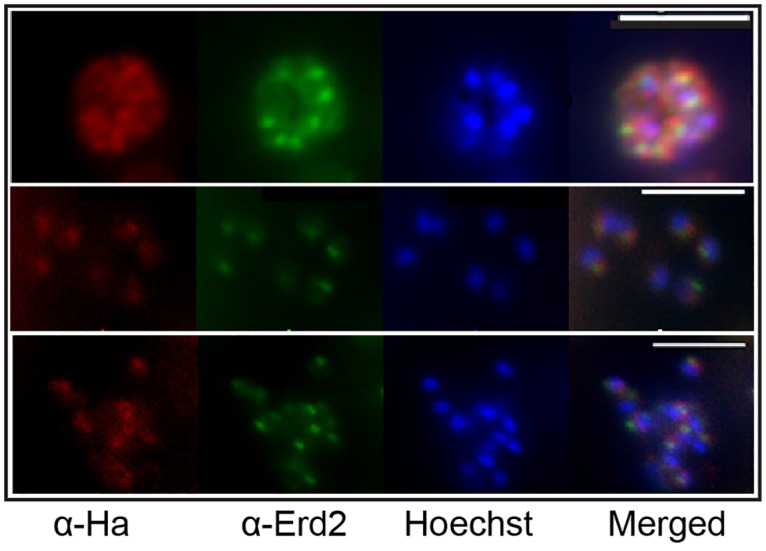
*Pb*S2P shows partial co-localisation with the cis-Golgi marker ERD2. (A) Double labelling IFA of *P*. *berghei* schizont cultures using α-HA (3F10) for detection of *Pb*S2P (red) and α-ERD2 as a Golgi marker (green) showing partial, or in some cases complete, co-localisation. Nuclei are stained with Hoechst (blue). Scale bar 5 μM.

At the protein level, a phosphorylated peptide from *P*. *falciparum* S2P has been identified in schizont phosphoproteome studies, albeit at very low levels [43, 44]. To obtain a better understanding on the localisation and expression of *Pb*S2P we tagged the endogenous gene at the C-terminus with a triple HA epitope (3HA) by double crossover homologous recombination (S2A Fig). *In vivo* cloning by limiting dilution resulted in 2 clonal lines, which were confirmed by PCR genotyping (S2B Fig). Whole schizont extracts were used for Western blot analysis to confirm expression of *Pb*S2PHA (Fig 2B). The tagged protein migrated faster on the SDS-PAGE gel, at ~35 kDa instead of 45 kDa, the expected molecular weight. This could be due to the seven hydrophobic transmembrane segments of the protein. IFA analysis in schizonts/merozoites and ookinetes revealed localisation of *Pb*S2PHA in proximity to the nucleus (Fig 2C) and double-labelling experiments with the Golgi marker ERD2 [45] in schizont cultures revealed close association of S2P with ERD2, although there was not complete overlap of the signals (Fig 3).

A signal was detected in mature oocysts (d12-d14 after mosquito infection; Fig 2C) and in mature salivary gland sporozoites. In the latter, the protease was localised close to the nucleus (Fig 2C). Together, our spatio-temporal analysis of *Pb*S2P protein profiling revealed localization adjacent to the nucleus in invasive stages and expression in most parasite stages.

### Targeted disruption of *PbS2P* does not affect gametogenesis nor sporogonic development

To investigate the function of *PbS2P*, we created a construct to delete the target gene in a GFP-expressing *P*. *berghei* line [26, 46]. This construct, containing 5’ and 3’ regions of the *PbS2P* ORF as targeting sequences, is expected to inactivate the gene by double crossover homologous recombination and gene replacement with the *Toxoplasma gondii dhfr/ts* positive selection cassette (S2C Fig). We anticipated that the gene would be refractory to deletion as it is essential in other organisms. Surprisingly, recombinant parasite populations were readily obtained after selection with pyrimethamine and subsequent cloning. *PbS2P* disruption of the gene was verified by PCR genotyping and Southern blot (S2D and S2E Fig). We conclude that *PbS2P* is not essential for intra-erythrocytic development of the parasite.

**Fig 4:**
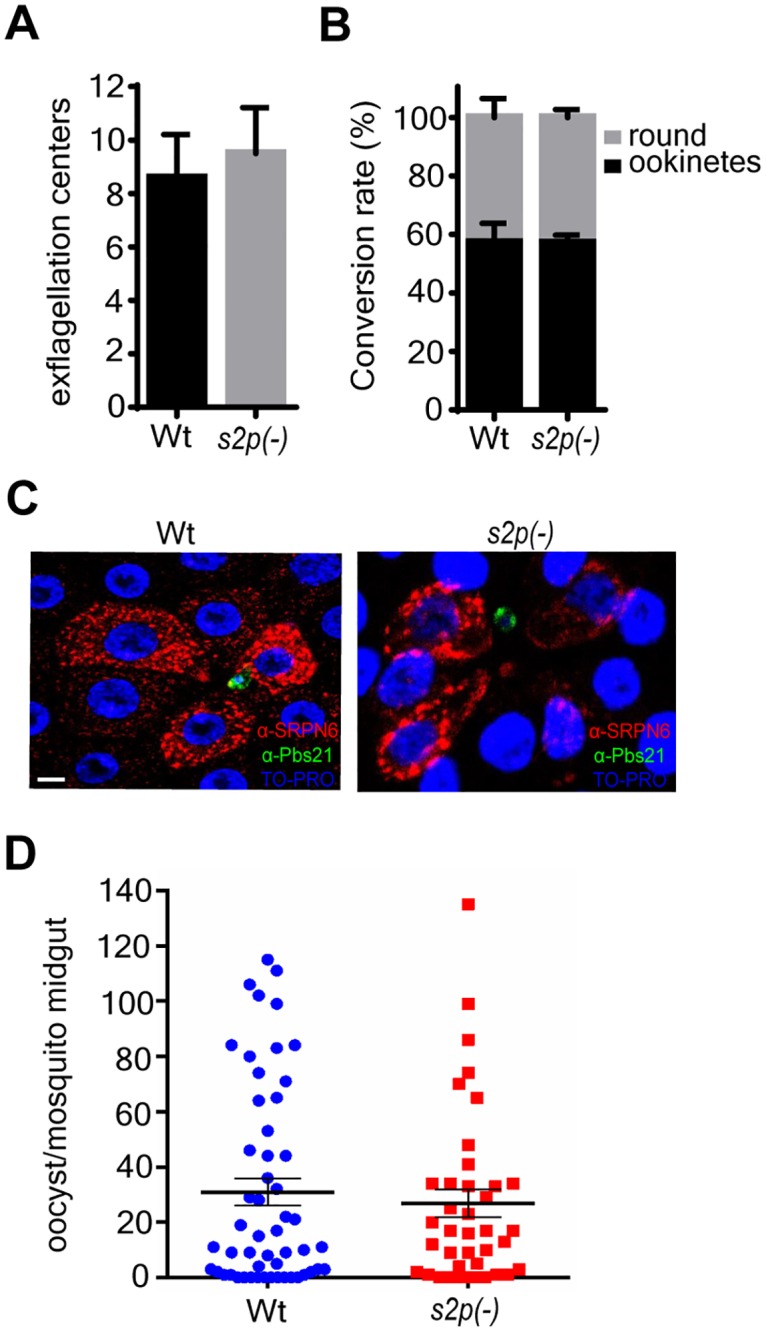
*s2p(-)* parasites show no defects in sexual development nor sporogony. (A) Exflagellation assay showing formation of exflagellation centres. Mean values (±SD) from three independent experiments are shown. Differences were non-significant (Mann-Whitney test). (B) Ookinete conversion rates of WT and *s2p(-)* parasites after staining with an antibody against the surface antigen P28 and enumeration of ookinetes, zygotes and macrogametes. Shown are mean values (±SD) from three independent experiments. Differences were non-significant (2way-ANOVA). (C) Immunofluorescence analysis of *Anopheles gambiae* epithelia sheets infected with WT or *s2p(-)* parasites. Both strains induce an epithelial response as shown by the SRPN6 antibody (red). Ookinetes are stained with an antibody against surface protein P28 (green) and nuclei are stained with TO-PRO 3 (blue). Scale bar 10 μM. (D) Oocyst numbers of *s2p(-)* strain compared to the parental WT line after standard membrane feeding assay of *An*. *gambiae* mosquitoes from two independent experiments. Black bars show mean values (±SEM). Differences were non-significant (Mann-Whitney test).

We continued our analysis by examining the effect of *PbS2P* depletion throughout the life cycle. Mice were infected with a high inoculation dose of 1x10^6^ *s2p(-)* or WT iRBCs to study gametocyte formation, which can be quantified by enumerating exflagellation centre formation representing activated male gametes. The propensity to form viable male gametes was indistinguishable between WT and *s2p(-)* parasites (Fig 4A).

Next, we quantified the gamete/ookinete conversion rate from *in vitro* ookinete cultures by staining gametes, zygotes and ookinetes with an antibody against the surface protein P28 [30]. Zygotes can only form when female gametes are viable and fertilized, providing a robust read-out for female gamete function. Again, conversion rates were identical between WT and *s2p(-)* parasites (Fig 4B). Furthermore, *s2p(-)* parasites do not exhibit any defect in penetrating the midgut epithelium of *A*. *gambiae* mosquitoes and induce similar epithelial responses as indicated by SRPN6 staining (Fig 4C). Moreover, standard membrane feeding assay did not reveal any difference in the number or morphology of developing oocysts (Fig 4D).

To explore a potential role of *PbS2P* in pre-erythrocytic stages we performed an initial experiment and showed that infected *A*. *gambiae* mosquitoes, which display a relatively low vectorial capacity for *P*. *berghei*, were able to transmit *s2p(-)* parasites to C57BL/6 mice. Upon exposure to 20 mosquitoes, 2/2 WT and 2/2 *s2p(-)* mice became blood-smear positive. However, we noticed a substantial, two day delay in the prepatency of mice infected with *s2p(-)* parasites, which became patent on day 7 in comparison to day 5 in WT-infected mice, suggesting a defect in the pre-erythrocytic phase of the parasite life cycle.

### Ablation of *Pb*S2P impairs liver stage development and intraerythrocytic growth

**Fig 5:**
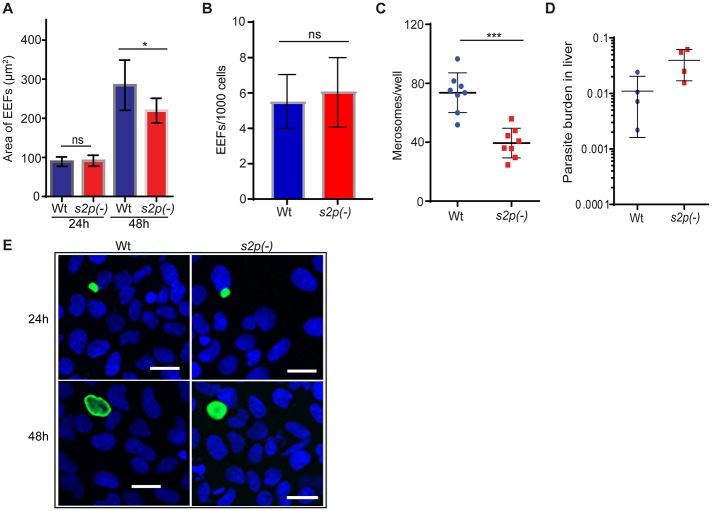
Growth of *s2p(-)* parasites is affected in late liver stages. (A) Sizes of WT and *s2p(-)* liver stages from cultured hepatoma cells, 24 and 48h after infection. The area covered by *Pb*HSP70 was measured and values are displayed as mean (±SD) of two independent experiments done in triplicate and quadruplicate wells, respectively. *, *P*<0.05 (two-tailed, unpaired Student's *t*-test). (B) Quantification of EEFs per 1000 cells at 24 hr after infection. Values represent mean numbers (± SD) from two independent experiments done in triplicate and quadruplicate wells respectively. ns, not significant (unpaired Student's *t*-test). (C) Merosomes from culture supernatants of WT and *s2p(-)* infected hepatoma cells were collected and enumerated in a Neubauer chamber. Mean values (± SD) from two independent experiments done in quadruplicate are shown. ***, *P*<0.001 (Mann-Whitney test). (D) Relative parasite loads in mouse livers as determined by qRT-PCR analysis after intravenous injection of 10,000 WT or *s2p(-)* sporozoites into C57BL/6 mice (n = 4 each). Livers were harvested 40h later and results show relative expression of *Pb*18S rRNA, normalised to mouse *GAPDH*. Differences were non-significant (Mann-Whitney test). (E) Representative images of EEFs at the time points indicated. Intracellular parasites were labelled with monoclonal mouse anti-*Pb*HSP70 followed by Alexa 488-conjugated secondary antibodies and Hoechst staining. Scale bar 30 μM.

In order to further examine this defect and study the growth of *s2p(-)* parasites in hepatocytes we analysed liver stage development *in vitro* and *in vivo*. *A*. *stephensi* mosquitoes, which are highly permissive for *P*. *berghei*, were allowed to feed on NMRI mice infected with WT or *s2p(-)* parasites at similar parasitaemia/gametocytaemia. 19 days later, salivary gland sporozoites from WT and *s2p(-)* -infected mosquitoes were isolated and enumerated. This analysis revealed similar sporozoite numbers in both parasite lines. In WT-infected *A*. *stephensi* mosquitoes an average of 13,500 (±3,100) sporozoites were enumerated, in comparison to *s2p(-)* displaying an average of 13,200 (±3,000), in four independent experiments. Next, we infected Huh7 hepatoma cells and monitored development of exo-erythrocytic forms (EEFs) 24h and 48h after sporozoite infection (Fig 5A).

The *s2p(-)* parasites showed a small, albeit significant, size reduction at 48h (Fig 5A and 5E) but the number of EEFs was very similar (Fig 5B). We then enumerated merosomes, which are membrane-bound vesicles containing first generation merozoites (Fig 5C). *s2p(-)-*infected hepatoma cells produced two-fold fewer merosomes as compared to WT. The above results suggested a reduced growth rate of *s2p(-)* parasites in late liver stage development, in good agreement with a ~2-fold upregulation of *PbS2P* transcripts 48h after infection as compared to 24h (Fig 2A).

To corroborate these observations, we injected C57BL/6 mice intravenously with 10,000 WT or *s2p(-)* salivary gland sporozoites and removed the livers 40 h later to determine parasite loads *in vivo* by qRT-PCR (Fig 5D). We observed no defects in the infection of the mouse liver by *s2p(-)* parasites, indicating normal infection and supporting the notion that the growth defect commences only in the final stages of liver stage development.

**Fig 6:**
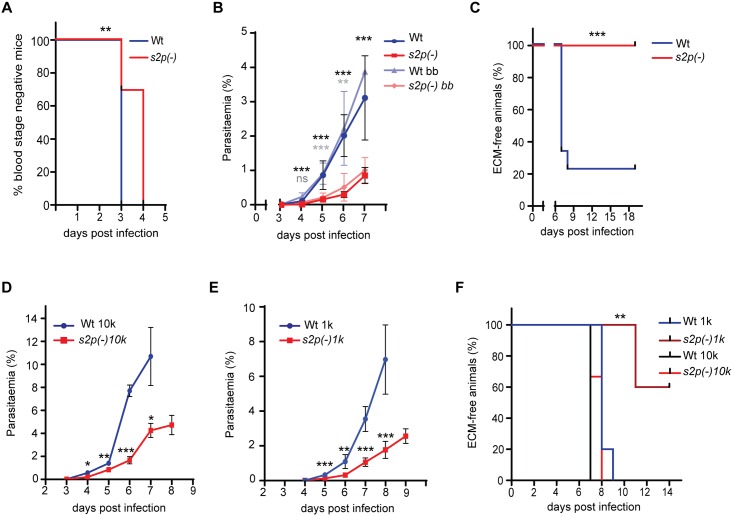
*s2p(-)* parasites show delayed transmission and growth, resulting in reduced virulence. (A) Kaplan-Meier analysis of time to patency after inoculation of 10,000 WT or *s2p(-)* salivary gland sporozoites into C57BL/6 mice (WT n = 9, *s2p(-)* n = 13). **, *P* < 0.01 (Log rank [Mantel-Cox] test). (B) Blood stage growth curve of the same mice showing the difference in growth rate of WT *vs*. *s2p(-)* parasites, alongside blood stage development of mice infected through bite back from infected *A*. *stephensi* mosquitoes (Wt bb *vs*. *s2p(-)* bb). In all cases mean values (± SD) are shown. **P < 0.01, ***P < 0.001 (Multiple t-test comparison). Significance is shown as follows: Black stars: Sporozoite injection experiment, Grey stars: Bite back experiment. (C) Kaplan-Meier curve of mice developing experimental cerebral malaria (ECM) over time after injection of 10,000 WT or *s2p(-)* salivary gland sporozoites (WT n = 9, *s2p(-)* n = 13). ***, *P* < 0.001 (Log rank [Mantel-Cox] test). (D, E) Parasitaemia levels of C57BL/6 mice after infection with (D) 10,000 (*n* = 3 each) or (E) 1,000 (*n* = 5 each) WT or *s2p(-)* iRBCs, respectively, as determined by Giemsa stained blood smears. *s2p(-)* parasites exhibit slower growth rates compared to the WT line. Mean values (± SD) are shown *P < 0.05 **P < 0.01, ***P < 0.001 (Multiple t-test comparison). (F) Kaplan-Meier curve of time to ECM development after patency. A one day delay in ECM symptoms was observed in mice infected with 10,000 *s2p(-)* iRBCs (*P* = 0.11 (Log rank [Mantel-Cox] test). Mice infected with 1,000 WT iRBC developed ECM symptoms at day 8 (4/5) and 9 (1/5), while 2/5 *s2p(-)* infected mice developed ECM at day 11 and 3/5 remained free of ECM symptoms. **P < 0.01 (Log rank [Mantel-Cox] test).

To further validate these results and the 2-day delay in prepatency observed in *A*. *gambiae* transmission experiments, we again injected 10,000 WT or *s2p(-)* salivary gland sporozoites from infected *A*. *stephensi* mosquitoes into C57BL/6 mice intravenously and determined prepatency. In WT-infected mice, blood stage parasites appeared 3 days after infection in all (9/9) mice, while in *s2p(-)* infected mice, the majority (9/13) of mice showed blood stage parasites one day later (Fig 6A).

To further support these data we next performed bite back experiments by exposure of naïve mice to six infected *A*. *stephensi* mosquitoes (*n = 5* for *s2p(-)*, *n* = 3 for WT). *s2p(-)*-infected mice displayed a mean prepatency of 3.4 days as compared to 3.0 days in WT-infected mice (3/3). When we monitored blood stage growth after natural sporozoite transmission by mosquito bite or controlled sporozoite inoculation, we consistently noticed a striking delay in the growth of *s2p(-)* parasites compared to WT (Fig 6B). Importantly, after controlled sporozoite injection, none of the *s2p(-)* infected mice exhibited symptoms of experimental cerebral malaria (ECM), which typically develop at day 7–9 after infection, as seen in most (7/9) WT-infected mice (Fig 6C).

The delay in blood stage growth was not observed in initial experiments where mice were infected with 1x10^6^ iRBCs. This prompted us to examine in more detail the asexual erythrocytic cycle in order to distinguish a pre-erythrocytic impairment from a defect in blood stage propagation. To this end, C57BL/6 were infected with 10,000 (*n* = 3 each) or 1,000 (*n* = 5 each) WT or *s2p(-)* iRBCs, and blood stage parasitaemia was monitored daily (Fig 6D and 6E). In both experiments mutant parasites did appear on the same day after infection as WT, but their growth rate was significantly slower. Mice infected with 10,000 *s2p(-)* iRBCs developed ECM symptoms one day later (on day 8 p.i.) as compared to WT-infected mice (on day 7 p.i.). Similarly, inoculation of 1,000 iRBCs resulted in a difference on ECM development; all WT infected mice developed ECM, but only 2/5 of the *s2p(-)*infected (Fig 6F). In addition, onset of ECM symptoms for the latter two *s2p(-)* infected mice was delayed by 3 days, *i*.*e*. on day 11 p.i *vs*. day 8 p.i. in WT-infected mice (Fig 6F), confirming the attenuation of virulence observed after sporozoite inoculation (Fig 6A).

In conclusion, our results suggest that ablation of *PbS2P* affects both late liver stage and asexual red blood cell development, resulting in virulence attenuation during blood infection.

## Discussion

The *Plasmodium* genome encodes more than 100 proteases [47], many of which have been largely unexplored in terms of their biological function and their potential as putative drug targets. In this work, we have examined the *in vivo* function and spatio-temporal profile of *Plasmodium berghei* S2P, a metalloprotease of the M50 family. Previous studies have identified members of this protease family in almost all evolutionary lineages. They all share a conserved three transmembrane domain core structure and metal-atom coordinating residues. But there is wide variation amongst S2P proteases regarding the total number of transmembrane domains. S2P contain intriguing features in their primary sequences including a variable serine repeat which function is unknown and a Cys rich region similar to PDZ domains [2]. 3D homology modelling and amino acid sequence analysis revealed that *PbS2P* does not contain these additional features, but like other S2Ps clearly exhibits hydrophobicity in the residues surrounding the conserved HEXXH motif. These observations suggest that *Plasmodium* S2Ps might be more closely related to the M50B subfamily which includes the SpolVFB sporulation factor and other plant and bacteria M50 proteases.

*PbS2P* is expressed in all invasive stages (merozoites, ookinetes and sporozoites) and localises in close vicinity to the nucleus. In most eukaryotic organisms S2P localises to the Golgi. A notable exception includes plants, some of which have multiple S2P proteases, where the different homologues reside in the chloroplast [48]. Most members of the *Apicomplexa* phylum, including *Plasmodium* parasites, contain a non-photosynthetic plastid termed apicoplast being located close to the nucleus. Further studies in *P*. *falciparum*, where a wide array of antibodies are available for localisation studies might provide a conclusive answer regarding the localisation of S2P in *Plasmodium* parasites.

Genetic ablation of *PbS2P*, in contrast to our predictions, did not result in a lethal phenotype at any developmental stage. From our phenotypic analysis it is evident that absence of *PbS2P* has a fitness cost for the parasite as documented by virulence attenuation due to reduced growth in both hepatic and blood stages. The growth delay probably commences at or close to 48h after liver infection, when *pbs2p* transcript levels increase two-fold compared to 24h EEFs (Fig 2A). This leads to a reduced number of merosomes and, in most cases, a delay in patency. But it is also clear that *s2p(-)* parasites have a profound defect in blood stage development *per se*. This defect can be masked when using high inoculum of infected parasites (10^6^), but not in infections with a lower inoculum. An obvious question that arises is if this defect occurs in schizont stages since expression levels of *PbS2P* specifically increase in the later stages of the intraerythrocytic cycle. In the murine malaria model functional assays for schizogony are limited, since *P*. *berghei* schizonts do not rupture *in vitro*, while *in vivo* they get sequestered to the spleen and the liver [49].

The virulence attenuation and growth delay in *s2p(-)* parasites is clearly reminiscent of the phenotypes observed in S2P null mutants of certain bacteria and fungi. Prominent examples include *M*. *tuberculosis* were ablation of *Rip1* results in a hundred-fold reduction in bacteria titres in lung of mice during acute infection [50] and *E*. *faecalis* where mutant bacteria are severely attenuated in an endocarditis rabbit model [51]. In a similar manner, the homologue of *S2P* in *C*. *neoformans* is required for virulence in mice and deletion of the gene leads to a drastic decrease of survival in the presence of azole drugs [52].

The absence of a lethal phenotype does not exclude essential functions of this protease in both blood and liver stages, which might be complemented by another protease. A well-documented example involving S2P is in *Drosophila melanogaster*. Flies rely on SREBP for survival, which is processed by S2P. In the absence of the protease, SREBP is cleaved by a caspase (*Drice*) and this alternate processing is sufficient for the flies to survive [53]. Caspases have not been yet identified in any *Plasmodium* species, which only encode three metacaspase-like proteases. Of note, *Plasmodium* parasites and other *Apicomplexa* encode in their genomes an M50B-like protease (PFAM13398). This class of proteases is specific to bacteria and plants and members have the HExxH motif but lack the NPDG motif. Instead, there is a conserved Gly downstream of the HExxH sequence and a conserved Asp residue which might be participating in active site formation alongside the two histidine residues. In *P*. *berghei*, gene PBANKA_1362500 encodes a putative M50B-like metalloprotease and studies on this gene might provide answers regarding any potential overlapping functions of these molecules.

An obvious emerging question concerns the precise role(s) and the physiological substrates of *PbS2P*. No homologues of ATF6 or SREBP have been identified in Apicomplexa parasites and current data suggest that *Plasmodium* parasites are devoid of bHLH or bZIP transcription factors, relying largely on a family of ApiAP2 transcription factors for gene regulation [54]. However, a small number of other transcriptions factors have been identified, such as Myb1 and PREBP [55, 56]. Of the 32 AP2 transcription factors annotated in *P*. *berghei* only one contains predicted transmembrane domains (PBANKA_1356000), and it could be a candidate substrate for *PbS2P*.

*Plasmodium* parasites lack the biosynthetic machinery to synthesize sterols and rely on host cells for lipid acquisition. Thus a potential role for *PbS2P* in interacting with host transcription factors that regulate cholesterol and lipid metabolism seems unlikely. Recent studies suggest that UPR in *Plasmodium* is regulated by the PERK–eIF2α pathway and by the upregulation of genes that belong to the AP2 family of transcription factors [57]. A role of the host UPR after hepatocyte infection has been recently suggested [58]. Downregulation of CREBH, a *bona fide* substrate of S2P, severely impairs liver infection, and it will be interesting to examine the effects of downregulation of the host S2P in infected liver cells. S2P in *Plasmodium* parasites might be activated in response to a completely different signal, such as oxidative stress. S2P has been recently shown to be involved in oxidative stress responses in mammalian cells, where ablation of S2P renders cells more susceptible to oxidative stress regulating expression of paraoxonase-2 (PON-2) [59]. Further studies will be needed to identify the signalling pathway that *Plasmodium* S2P is involved in as well as that of related Apicomplexa S2Ps.

In conclusion, we have provided evidence that *PbS2P* is expressed in invasive and sporogonic stages of the parasite and ablation of it results in reduced hepatocyte and intraerythrocytic development of the malaria parasite. This fitness cost for the parasite results in virulence attenuation but the underlying mechanism is still elusive. Studies of related proteins (M50B-like) and monitoring the growth of *s2p(-)* parasites under different stress conditions will provide evidence for the signalling mechanism controlled by S2P and ultimately resolve whether *Plasmodium* M50 proteases are candidate targets for evidence-based malaria intervention strategies.

## Supporting Information

S1 Fig Multiple sequence alignment of S2P orthologues. Multiple alignment of the protein sequences of S2P orthologues from *Plasmodium* species and related apicomplexan parasites alongside S2Ps from other prokaryotic and eukaryotic organisms. % Identity of S2Ps between different organisms is also shown. Uniprot (www.uniprot.org) entry numbers for each S2P are as follows: *P*. *berghei*: Q4YUC6, *P*. *chabaudi chabaudi*: A0A077TQD2, *P*. *yoelii*: A0A077YAR7, *P*. *falciparum*: Q8IEQ9, *P*. *vivax*: A5JZ48, *P*. *knowlesi*: B3LBL7, *P*. *cynomolgi*: K6UMM1, *P*. *reichenowi*: A0A060S248, *T*. *gondii*: A0A125YNX6, *N*. *caninum*: F0VMA1, *E*. *tenella*: U6KY56, *H*. *hammondi*: A0A074SWV8, *E*. *coli*: P0AEH1, *M*. *tuberculosis*: P9WHS3, *B*. *subtilis*: O31754, *A*. *thaliana*: F4JUU5, *D*. *melanogaster*: Q7JZ56, *H*. *sapiens*: O43462. Sequence alignment was done with T-Coffee from the EMBL_EBI server and editing with ESPript 3.0.
(PDF)

S2 Fig Strategy for the generation of transgenic *PbS2PHA* and *s2p(-) P*. *berghei* parasites. (a) Schematic representation of the gene targeting strategy used for the tagging of pbs2p with a triple HA tag (3HA tag) using PlasmoGEM vector. Upon recombination pbs2p is tagged with a 3HA tag and the endogenous pbs2p 3’ UTR is replaced with the pbdhfr-ts 3’ UTR. Wild type specific (F1-F2) and replace-ment specific primers (F1-F3) are indicated by black arrows. The vector was digested with NotI prior to transfection. (b) PCR genotypic analysis of the derived transgenic clones (cl1 and cl2). The primer pair F1-F2 amplifies the wt locus containing the endogenous pbs2p 3’ UTR. Failure to amplify this 430 bp fragment, indicates absence of the wt locus from genomic DNA isolated from all clones. Primer pair F1-F3 amplifies a fragment corresponding to the replaced locus. (c) Schematic representation of the replacement strategy used to generate *s2p(-)* parasites. Upon double cross-over recombination, part of the gene is replaced with the selectable marker tgdhfr/ts. Restriction enzymes KpnI and XbaI have been used to linearise the vector. (d) PCR genotyping using primer pairs KD5For-L695 and KD3Rev-L665 for 5’ and 3’ integration respectively and KD5For-KD3Rev to show absence of the wt locus in the obtained clones. (e) Southern blot analysis of genomic DNA isolated from WT and s2p(-) parasites, using a biotin labelled probe for pbs2p. The probe hybridizes to a 3.3 kb fragment in WT and a 1.9 kb fragment in s2p(-) parasites.
(TIF)

S1 Table Primers used in this study. (PDF)
