# Supplementary material for: Targeted Deletion of a Plasmodium Site-2 Protease Impairs Life Cycle Progression in the Mammalian Host
Source: PLoS One. 2017 Jan 20;12(1):e0170260. doi: 10.1371/journal.pone.0170260 (PMC5249076; doi:10.1371/journal.pone.0170260)
Supplement: S1 Fig — Multiple alignment of the protein sequences of S2P orthologues from Plasmodium species and related apicomplexan parasites alongside S2Ps from other prokaryotic and eukaryotic organisms. % Identity of S2Ps between different organisms is also shown. Uniprot (www.uniprot.org) entry numbers for each S2P are as follows: P. berghei: Q4YUC6, P. chabaudi chabaudi: A0A077TQD2, P. yoelii: A0A077YAR7, P. falciparum: Q8IEQ9, P. vivax: A5JZ48, P. knowlesi: B3LBL7, P. cynomolgi: K6UMM1, P. reichenowi: A0A060S248, T. gondii: A0A125YNX6, N. caninum: F0VMA1, E. tenella: U6KY56, H. hammondi: A0A074SWV8, E. coli: P0AEH1, M. tuberculosis: P9WHS3, B. subtilis: O31754, A. thaliana: F4JUU5, D. melanogaster: Q7JZ56, H. sapiens: O43462. Sequence alignment was done with T-Coffee from the EMBL_EBI server and editing with ESPript 3.0. (PDF) [file pone.0170260.s001.pdf]

```
               1                                          10        20
P.berghei      MTSRNIISY..................................FNDVQTEKKQLSSNWL
P.vivax        MDPKNHVLF..................................CSNALMEHRRVASNFL
P.yoelii       MTSRNIISY..................................FNNVQTEKKQLSSNWL
P.chabaudi     MTPRNIISY..................................FNDVQTEKRQLSSNWL
P.cynomolgi    MDPKNNMFF..................................CSNTLMEHRRVASNFL
P.falciparum   MGSRNKMLL..................................YNNALMEHKRLASSFL
P.knowlesi     MDPKNNMFF..................................CSNTRMENRRLGSNYL
P.reichenowi   MGSRNKMLL..................................YNNALMEHKRLASSFL
T.gondii       MSGAVRIDARG...MIIREGEDPELGRPLRSAAESHATDLR..ASGRTLEQRQVSTRSS
N.caninum      MAGSVRIDARG...MPF..........................................
H.hammondi     MSGAVRIDARG...MIIREGQDPELGRPLHSAPESHAADLQ..ASGRTLDQRQVSTRTS
E.tenella      MAPQPQGGG....PRITA.TG...............DIIWP..GPNPSTRQQGLALPLA
A.thaliana     MEISGRRMRRF...RM...............................RFRRDHLTGG.E
E.coli         MLSFL......................................................
M.tuberculosis MMFV.......................................................
B.subtilis     MFVN.......................................................
H.sapiens      MIPVSLVVVVVGGWTVVY.LTD....LVLK......SSVYFKHSYEDWLENNGLSISPF
D.melanogaster MDPFVFFIVLASLYGVLY.FFD....RFFK......SCMHY..PYDAFLKNTGLSINFM

                   30                                    40
P.berghei      LIHNEKSYWDHKD...........................QS.................
P.vivax        PTYREKSYDYERH...........................QD.................
P.yoelii       LIDNEKSYWDYKR...........................QS.................
P.chabaudi     LIHNEKSYQDYKH...........................QS.................
P.cynomolgi    SMSREKSYAYERH...........................QD.................
P.falciparum   SVYKEKAYNYEKC...........................QN.................
P.knowlesi     YMWREKSYDYEKH...........................PD.................
P.reichenowi   SVYKEKGYNYEKC...........................QN.................
T.gondii       .......QPRGSIL.......GCLRRTFVRPFCCFPDAAG...........AR.....P
N.caninum      ................................CCFPDAAA...........SR.....P
H.hammondi     .......RPRGSIL.......GCLRRTFVRPFCCFPDAAA...........AR.....P
E.tenella      .....AGGQQQQQ..............................................
A.thaliana     NIENEAS.........................CCYCDLKISNFNEPIFRLGRRFSGVLK
E.coli         ...........................................................
M.tuberculosis ...........................................................
B.subtilis     ...........................................................
H.sapiens      HIRWQTAVFNRAFYSWGRRKA....RML..............................Y
D.melanogaster SLHWHTSAFNRTLLRWGSAGNSCTRRVM..............................I

                                 50        60        70        80
P.berghei      .........YLSFILSLLPLFLISVIHLSIILNGYYIGHLFLTISYLLS..........
P.vivax        .........VVSLILSMLPLLMTSVIHLLIKTNGYYIGNLFLMITYLFS..........
P.yoelii       .........YLSFILSLLPLFLISVIHLSIILNGYYIGHLFLTISYLLS..........
P.chabaudi     .........YLSLILSLLPLFLISVIHLSITMNGYYIGHLFLTISYLLS..........
P.cynomolgi    .........GVSFILSLFPLLIISGVHLWIKTNGYYIGNLFLMITYFFS..........
P.falciparum   .........CLSYFLSLVPLLIVGGIHLFIKMNGYYIGNLFLTITYLFS..........
P.knowlesi     .........VLSFILSMVPLLIVIVVHLLINTNGYYIGNLFLMITYLFS..........
P.reichenowi   .........CLSYFLSLVPLLIVGGIHLFIKMNGYYIGNLFLTITYLFS..........
T.gondii       EGAYTGF..WLPPILGLAPIFWLTI...................FAA.I..........
N.caninum      EGAYDAC..WVPPILGLAPVFWLTI...................FAT.I..........
H.hammondi     EGAYTGF..WLPPFLGLAPIFWLTI...................FAA.I..........
E.tenella      ...QQ....G.................................................
A.thaliana     VWFSIGLGF......GVASLIL..........................V..........
E.coli         ...........................................................
M.tuberculosis ...........................................................
B.subtilis     ...........................................................
H.sapiens      QWFNFGMVFGVIAMF....SSFFLL...................GKT.LMQTLAQMMAD
D.melanogaster TSFNV....GVLVTFSLLPIGLILL...................IAT.I..........
```

```
                                                                      90
                                                                       .
P.berghei        .................................ICFFIIYFYNSYT.............
P.vivax          .................................FFFFILYYYNSYA.............
P.yoelii         .................................ICFFILYFYNSYT.............
P.chabaudi       .................................ICFFILYFYNSYT.............
P.cynomolgi      .................................FFFFILYYYNSYA.............
P.falciparum     .................................FFFFILYYYNSYI.............
P.knowlesi       .................................FFFFILYYYNSYA.............
P.reichenowi     .................................FFFFILYYYNSYI.............
T.gondii         .................................GCGILAQFYWQPL.............
N.caninum        .................................GFGVAAHFYWQPV.............
H.hammondi       .................................GCGILAHFYWQPL.............
E.tenella        ............................................IA.............
A.thaliana       .................................TVFLLLQFHSNPLFSNRLTSAVFGFS
E.coli           ...........................................................
M.tuberculosis   ...........................................................
B.subtilis       ...........................................................
H.sapiens        SPSSYSSSSSSSSSSSSSSSSSSSSSSSLHNEQVLQVVVPGINLPV.............
D.melanogaster   ....FSSGEQD.........SSSSVSSPVGVPVQLEILLPGVNLPL.............

                              100       110       120       130       140
                                .         .         .         .         .
P.berghei        .......IFILFVFFSFIISLCLHEFAHALVAYKYGDITMVYK.GYLYLDIFNYLDIFH
P.vivax          .......VFVLFVFSSFFISLCLHEFAHALVAYKYGDITMVYK.GYLYLDIANYLDIFH
P.yoelii         .......IFVLFVFLSFIISLCLHEFAHALVAYKYGDITMVYK.GYLYLDILNYLDIFH
P.chabaudi       .......IFVLFVFFSFIISLCLHEFSHALVAYKYGDITMVYK.GYLYLDILNYLDIFH
P.cynomolgi      .......VFVLFVFFSFFISLCLHEFAHALVAYKYGDITMVYK.GYLYLDIVNYLDIFH
P.falciparum     .......IFVLFVFFAFLIYLCLHEFAHALVAYKYGDITMIYK.GYLYLDILNYLDIFH
P.knowlesi       .......VFVLFVFFSFFISLCLHEFAHALVAYKYGDITMVYK.GYLYLDIVNYLDIFH
P.reichenowi     .......IFVLFVFFAFLIYLCLHEFAHALVAYKYGDITMIYK.GYLYLDILNYLDIFH
T.gondii         .......LLTGLVVLGYILSVALHEFAHAATAFKGGDESVVYS.GYLTLDYLRYTSPLF
N.caninum        .......ILTGFVVFGYILSVALHEFAHAATAFKGGDESVVYS.GYLTLDYLRYTSPLF
H.hammondi       .......LLTGLVVLGYILSVALHEFAHAATAFKGGDESVVYS.GYLTLDYLRYTSPLF
E.tenella        .......CVSAFTVLLCLIGLCLHEFAHAATAYQAGVEGVAES.GYLTCDLIRYVGILD
A.thaliana       PSTRVSLSGIAYVLVSTVITVSVHELGHALAAASEGIQM.EYI.AVFIA..........
E.coli           .......WDLASFIVALGVLITVHEFGHFWVARRCGVRVERFSIGFG.KALWRRTDKLG
M.tuberculosis   .......TGIVLFALAILISVALHECGHMWVARRTGMKVRRYFVGFG.PTLWSTR.RGE
B.subtilis       ........TVIAFIIIFGTLVFFHELGHLLLAQRAGILCREFAIGFG.PKIFSF.KKNE
H.sapiens        .......NQLTYFFTAVLISGVVHEIGHGIAAIREQVRFNGFGI.FL............
D.melanogaster   .......EEIGYYITTLVLCLVVHEMGHALAAVMEDVPVTGFGIKFI............

                    150
                     .
P.berghei        TLIIPLITLFITGF.............................................
P.vivax          TLIIPSITLFITGF.............................................
P.yoelii         TLIIPLITLFITGF.............................................
P.chabaudi       TLVIPLVTLFITGF.............................................
P.cynomolgi      TLIIPCIMLLTTGF.............................................
P.falciparum     TLIIPLITLFITGF.............................................
P.knowlesi       TLVIPLVTLLTTGF.............................................
P.reichenowi     TLIIPLITLFITGF.............................................
T.gondii         SLGLPLLFLLLGNV.............................................
N.caninum        SLGLPLLFLLLGNV.............................................
H.hammondi       SLGLPLLFLLLGNV.............................................
E.tenella        VLIF.VFFVATGGI.............................................
A.thaliana       .......A..................IFPG.............................
E.coli           TEYV.IALIPLGGYVKMLD......................ERAEPVVPE.........
M.tuberculosis   TEYG.VKAVPLGGFCDIAG......................M.................
B.subtilis       TVYT.IRLLPVGGFVRMAGEDPEMIEVKPGYTVGLLFNKEDQVEKVIINQKEKYPDALV
H.sapiens        ......FIIYPGAFVDLFT........................................
D.melanogaster   ......FCL.PLAYTELSH........................................
```

```
                                                         160       170
                                                           .         .
P.berghei       ....................................GIPGNLYWL..QLHFI..RSRFQ
P.vivax         ....................................GLPGNLYWI..QLHFI..RSRFQ
P.yoelii        ....................................GIPGNLYWL..QLHFI..RSRFQ
P.chabaudi      ....................................GIPGNLYWL..QLHFI..RSRFQ
P.cynomolgi     ....................................GLPGNLYWL..QLHFI..RSRFQ
P.falciparum    ....................................GIPGNLYWL..QLHFI..RSRFQ
P.knowlesi      ....................................GLPGNLYWL..QLHFI..RSRFQ
P.reichenowi    ....................................GIPGNLYWL..QLHFI..RSRFQ
T.gondii        ....................................ALPGAAVLI..QHENL..RGAQW
N.caninum       ....................................ALPGAAVTI..QHSSL..RGAKW
H.hammondi      ....................................ALPGAAVMI..QHENL..RGAHW
E.tenella       ....................................VLPGGRVNI..DERAV..RSRGW
A.thaliana      ........................................GLVAF..DNDVLQSLPSFN
E.coli          ..............................................LRHHAFNNKSVGQ
M.tuberculosis  .....................................TPVEELDPDERDRAMYKQATWK
B.subtilis      IEVETADLEHDMKITGYEQGKEDELSSFTVSETSFFIVDGEEVQIAPYNRQFGSKPVWQ
H.sapiens       .................................................THLQLISPVQ
D.melanogaster  .................................................DHLNSLRWFR

                180       190
                  .         .
P.berghei       LSFIFLSGPISDILYILLFVFF.....................................
P.vivax         LSFIYLSGPISDILCILLIVFF.....................................
P.yoelii        LSFIFLSGPISDILYILLFVFF.....................................
P.chabaudi      LSFIFLSGPISDILYILLFVSF.....................................
P.cynomolgi     LSFIYLSGPISDILCILVIVFF.....................................
P.falciparum    LSFIFLSGPLSDILYILFIVFF.....................................
P.knowlesi      MSFIYLSGPISDILCILVIVFF.....................................
P.reichenowi    LSFIFLSGPLSDILYILFIVFF.....................................
T.gondii        RTLTALAGPLATFLSGLLFSGL.....................................
N.caninum       KTLTALAGPLANLLCGFLFSGL.....................................
H.hammondi      RTLTALAGPLATFLSGLLFSGL.....................................
E.tenella       KTAIALAGPATNLLLGMLGSFC.....................................
A.thaliana      ALRIYCAGIWHNAVFCALCVFALFLLPVMLSP............FYKHGESLTVVDVP.
E.coli          RAAIIAAGPVANFIFAIFAYWLVFI............IGVP.GV......RPVVGEIAA
M.tuberculosis  RVAVLFAGPGMNLAICLVLIYAIALV...........WGLP.NL......HPPTRAVIG
B.subtilis      RIKAIAAGPIMNFILAYVILVMLGLI...........QGVP.SN......EPMLGQLT.
H.sapiens       QLRIFCAGIWHNFVLALLGILALVLLPVILLPFYYTGVGVL............ITEVA.
D.melanogaster  KLRVLCAGIWHNFVFAGVCY............LLISTVGITMSPLYAYNQHVVVTELT.

P.berghei       ...........................................................
P.vivax         ...........................................................
P.yoelii        ...........................................................
P.chabaudi      ...........................................................
P.cynomolgi     ...........................................................
P.falciparum    ...........................................................
P.knowlesi      ...........................................................
P.reichenowi    ...........................................................
T.gondii        ...........................................................
N.caninum       ...........................................................
H.hammondi      ...........................................................
E.tenella       ...........................................................
A.thaliana      ...........................................................
E.coli          NSIAAEAQIAPGTELKAVDGIETPDWDAVRLQLVDKIGDESTTITVAPFGSDQRRDVKL
M.tuberculosis  ETGCVAQEVS.................................................
B.subtilis      ...........................................................
H.sapiens       ...........................................................
D.melanogaster  ...........................................................
```

```
P.berghei       ...........................................................
P.vivax         ...........................................................
P.yoelii        ...........................................................
P.chabaudi      ...........................................................
P.cynomolgi     ...........................................................
P.falciparum    ...........................................................
P.knowlesi      ...........................................................
P.reichenowi    ...........................................................
T.gondii        ...........................................................
N.caninum       ...........................................................
H.hammondi      ...........................................................
E.tenella       ...........................................................
A.thaliana      ...................................SVSPLFG..YLSPGDVIVSLDGIQ
E.coli          DLRHWAFEPDKEDPVSSLGIRPRGPQIEPVLENVQPNS.AASKAGLQAGDRIVKVDGQP
M.tuberculosis  ...........................QGKLEQCTGPG.PAALAGIRSGDVVVKVGDTP
B.subtilis      ...................................DNG.RAAEAGLKEGDYIQSINGEK
H.sapiens       ...................................EDSPAIGPRGLFVGDLVTHLQDCP
D.melanogaster  ...................................RKSPLRGERGLQVDNQITQVNGCP
```

```
P.berghei       ...........................................................
P.vivax         ...........................................................
P.yoelii        ...........................................................
P.chabaudi      ...........................................................
P.cynomolgi     ...........................................................
P.falciparum    ...........................................................
P.knowlesi      ...........................................................
P.reichenowi    ...........................................................
T.gondii        ...........................................................
N.caninum       ...........................................................
H.hammondi      ...........................................................
E.tenella       ...........................................................
A.thaliana      VHKPSEWLELAAI.LDKENSKTSNGSLYLGGSRRFHHGKGYCVPI...........SLI
E.coli          LTQ...WVTFVML.VRDNPG....................KSLALEIERQGSPLSLTLI
M.tuberculosis  VSS...FDEMAAA.VRKSHG.....................SVPIVVERDGTAIVTYVD
B.subtilis      MRS...WTDIVSA.VKENPE....................KEMDVAVKRDNKTLHISVT
H.sapiens       VTNVQDWNECLDT.IAYEPQ..................IGYCISA...........STL
D.melanogaster  VNSEESWVTCLQNSLKL..K..................PGYCVSA...........DFV
```

```
P.berghei       ...........................................................
P.vivax         ...........................................................
P.yoelii        ...........................................................
P.chabaudi      ...........................................................
P.cynomolgi     ...........................................................
P.falciparum    ...........................................................
P.knowlesi      ...........................................................
P.reichenowi    ...........................................................
T.gondii        ...........................................................
N.caninum       ...........................................................
H.hammondi      ...........................................................
E.tenella       ...........................................................
A.thaliana      E.EGYK.....GKMVENQFVCPGDLT....AFRTMPC.......SNAAIR........E
E.coli          P..................................ES.K.......PG..NGKAIGFV.
M.tuberculosis  I..................................ESTQRWIPNGQGGELQPATVGAI.
B.subtilis      P..................................EAVK.......DE..NKKTIGRF.
H.sapiens       QQLSFPVRA..YKRLDGSTECCNNHSLTDVCF...SY.......RNNFNK..RL.....
D.melanogaster  Q.LNDESSAISHHSIDGQLQCCDELNPNVSCF...EV.......VEDANG..DVPVELP
```

```
P.berghei       ...........................................................
P.vivax         ...........................................................
P.yoelii        ...........................................................
P.chabaudi      ...........................................................
P.cynomolgi     ...........................................................
P.falciparum    ...........................................................
P.knowlesi      ...........................................................
P.reichenowi    ...........................................................
T.gondii        ...........................................................
N.caninum       ...........................................................
H.hammondi      ...........................................................
E.tenella       ...........................................................
A.thaliana      VSVCLD...........AKD.IVKLQKCGDGWVTTSDTDNQSDC.....VCPQGDLCLQ
E.coli          .GIEPKVIPLPDEYKVVRQYGPFNAI...........VEATDKTWQLMK..........
M.tuberculosis  .GVGAARVGP.......VRYGVFSAM...........PATFAVTGDLTVEVGK..ALA.
B.subtilis      .GSYA...P........TEKGVLSAV...........AYGATSTVDVTK..........
H.sapiens       .HTCLP...........ARK.AVEAT...........QV.CRTNKDCKKSSSSS.FCII
D.melanogaster  QHVCLN...........VRR.TLEEV...........SEHCSSG.....VCNEG.FCLR
```

```
P.berghei       ...........................................................
P.vivax         ...........................................................
P.yoelii        ...........................................................
P.chabaudi      ...........................................................
P.cynomolgi     ...........................................................
P.falciparum    ...........................................................
P.knowlesi      ...........................................................
P.reichenowi    ...........................................................
T.gondii        ...........................................................
N.caninum       ...........................................................
H.hammondi      ...........................................................
E.tenella       ...........................................................
A.thaliana      A.MQSPGVLWT.EITYKRT.SSQDCSRLGLDFNTSNCLGTFVFVGDLIAM...SHSVHL
E.coli          LT....VSMLG...KLITG.DV.................KLNNLSGPISIAKGAGMTAE
M.tuberculosis  ALPTK.VGALV...RAIGGGQR.............DPQTPISVVGASII...GGDTVDH
B.subtilis      AI....LTNLS...KLVTG.QF.................KLDMLSGPVGIYDMTDQVAK
H.sapiens       PS....LETHT.RLIKVKH.PPQ...............IDMLYVGHPLHL...HYTVSI
D.melanogaster  PL....IRNITAIMTFKRQ.NFR...........GEKLPPVIYVGHPWDV...TRTVEV
```

```
                   200         210         220           230       240       250
                     .           .           .             .         .         .
P.berghei       ...YN..MYTFFKNNIKFNVKPHPALF...ISLATSISFLVESFLLNICPILGFDGWGV
P.vivax         ...YN..FYSYVRNSKNLSVQPHSILF...ISLATAASFLVDSFLLNICPIFGFDGWGI
P.yoelii        ...YN..MYTFLKNNKKLNVNPHPALF...ISLATSISFLVESFLLNICPILGFDGWGV
P.chabaudi      ...YN..MYKFFKNNKKFNVKPHPALF...ISLATSISFLVESFLLNICPILGFDGWGI
P.cynomolgi     ...YN..FYAYFKNNKNLSVQPNSILF...ISLATAASFLVDSFLLNICPIFGFDGWGI
P.falciparum    ...YN..LYSYFKNHKNLNVQPHSILF...ISLATAVSFLVDSFLLNLCPILGFDGWGI
P.knowlesi      ...YN..FYSYFKNSKNLSAQPHSILL...ISLATAASVLVDSFLLNICPIFGFDGWGI
P.reichenowi    ...YN..LYSYFKNHKNLNVQPHSILF...ISLATAVSFLVDSFLLNLCPILGFDGWGI
T.gondii        ...LH..LSL.LSADYY.....YTILH...MGLACLIYFEAMSFIINMIPLPPLDGWAA
N.caninum       ...LR..LTL.LSVDYY.....YTVLH...MGLACLIYFESMSVIINMIPLPPLDGWAA
H.hammondi      ...LH..LSL.LSADYY.....YTVLH...MGLACLIYFEAMSFIINMIPLPPLDGWAA
E.tenella       ...VQ..VYIL.AASAN.....PSIIPLPIIGLICFIFLQFMSAVVNLIPFPP......
A.thaliana      TAYQPRWLFNFFGKSF......PNILE...RSLTCTFHVSLALVLLNSLPVYYLDGESI
E.coli          LGVVY..Y........................LPFLALISVNLGIINLFPLPVLDGGHL
M.tuberculosis  GLWVA..F........................WFFLAQLNLILAAINLLPLLPFDGGHI
B.subtilis      TGIVN..L........................FQFAAFLSINLGIVNLLPIPALDGGRL
H.sapiens       TSFIP..RFNFLSIDLP.....VVVET....FVKYLISLSGALAIVNAVPCFALDGQWI
D.melanogaster  SAFVP..RYSLLKAAWP.....DAWLL....LLKYNVVFSIGLALINAIPCFGFDGAHI
```

```
                      260          270          280       290      300
P.berghei      IEPYLPYFLNDIINEEIV..YNFLSY...VCPLLVFIYFNFIETKFFFFTKITNYILQD
P.vivax        VEPYLPYCVNSLINEEIV..NTYLSY...ICPLLVFIYFNFIETKYLFFTKIVNFILAR
P.yoelii       IEPYLPYFLNDLINEEIV..YNFLSY...VCPLLVFIYFNFIEAKFLFFTKITNYILQD
P.chabaudi     IEPYLPCFLNDLINEEIV..YNFLSY...VCPLLVFIYFNFIETKFLFFTKITNYILQD
P.cynomolgi    LEPYLPYCVNSLINEEIV..NTYLSY...ICPLMVFIYFNFIETKYLFFTKIVNFILAR
P.falciparum   LEPYLPYCLNNIINEEIV..YTYLSY...ICPLLVFIYFNFIEARYLFFTRIVNFILAR
P.knowlesi     LEPYLPYCLNTLINEEIV..NTYLSY...ICPLLVFIYFNFIETKYLFFTRIVNFILGR
P.reichenowi   LEPYLPYCLNNIINEEIV..YTYLSY...ICPLLVFIYFNFIETRYLFFTRIVNFILAR
T.gondii       LEPWLPHSC..FLKKAMD..DPTLQR...IVPLLVLAALFPVFARVPFFGHAVNVVAVT
N.caninum      LEPWLPRSC..FLRKAME..DPTLRR...ILPLFVLAALFPVFAKVPFFGHAVNAVAIT
H.hammondi     LEPWLPHSC..FLKKAMD..DPTLQR...IVPLLVLAALFPVFARVPFFGHAVNVVAVT
E.tenella      ...........................................................
A.thaliana     LESSLQSFTW..LSPRKK..KKALQVCLVGGSLLSFLAFFRIF................
E.coli         LFLAIEKIKGGPVSERVQD............................FCYRIGSILLVL
M.tuberculosis AVAVFERIRNMVRSARGKVAAAPVNY............LKLLPATYVVLVLVVGYMLLT
B.subtilis     LFLFIEAIRGKPINREKEA............................FVVFIGVAFLML
H.sapiens      LNSFLDATLTSVIGDNDV..KDLIGF.....................FILLGGSVLLAA
D.melanogaster TSTVIHSFLVGRVDQHAK..RDIISL.....................IITSVGSLLFAL
```

```
                   310       320                                330
P.berghei      LLGIAIDHAAFGADSFPTLYSYLK..........................KM
P.vivax        ILGIEISHVTIGVSAFPTLYAYLR..........................NV
P.yoelii       LLGIAIYHATFGADSFPTLYSYLK..........................KM
P.chabaudi     LLGIAIYHAAYGADSFPTLYSYLK..........................KL
P.cynomolgi    ILGIEISHVTIGVNAFPTLYAYLR..........................NV
P.falciparum   ILGIELGHVTNGVNTFPTLYSYLR..........................KI
P.knowlesi     ILGIEISHVTIGVNAFPTLFAYLR..........................NV
P.reichenowi   ILGIELGHVTNGVNTFPTLYSYLR..........................KI
T.gondii       IFRAPPDLTPLAMQYFAVPYSQWRYMHPIPVPVDMRRASVSSSEFLEDFLLQ
N.caninum      VFRAPSDLTPLAMQYFSMPYSQWRHMHPIPVPVDLRHAPA.STDFVEGFLLQ
H.hammondi     IFRAPPDLTPLAMQYFALPYSQWRYMHPIPVPVDMRRASVSSSEFLEGFLPQ
E.tenella      ..................................................LE
A.thaliana     LLGLP..........................................LSRRW
E.coli         LMGL.............ALFN..........................DFSRL
M.tuberculosis VT.A.............DLVN.........................PIRLFQ
B.subtilis     LMLV.............VTWN......................D..IQRLFL
H.sapiens      N.......V........TLGL.........................WMVTAR
D.melanogaster ALLK....V........AWLS.........................FLRPLL
```

% Identity to P. berghei

P. yoelii	94.68%
P. chabaudi	92.81%
P. reichenowi	72.18%
P. falciparum	71.87%
P. vivax	69.68%
P. cynomolgi	69.06%
P. knowlesi	68.12%
E. tenella	25.13%
T. gondii	24.06%
N. caninum	23.93%
H. hammondi	23.43%
A. thaliana	14.06%
D. melanogaster	12.50%
H. sapiens	11.56%
M. tuberculosis	10%
E. coli	8.43%
B. subtilis	7.81%
