# Supplementary figures and images for: Targeted Deletion of a Plasmodium Site-2 Protease Impairs Life Cycle Progression in the Mammalian Host
Source: PLoS One. 2017 Jan 20;12(1):e0170260. doi: 10.1371/journal.pone.0170260 (PMC5249076; doi:10.1371/journal.pone.0170260)

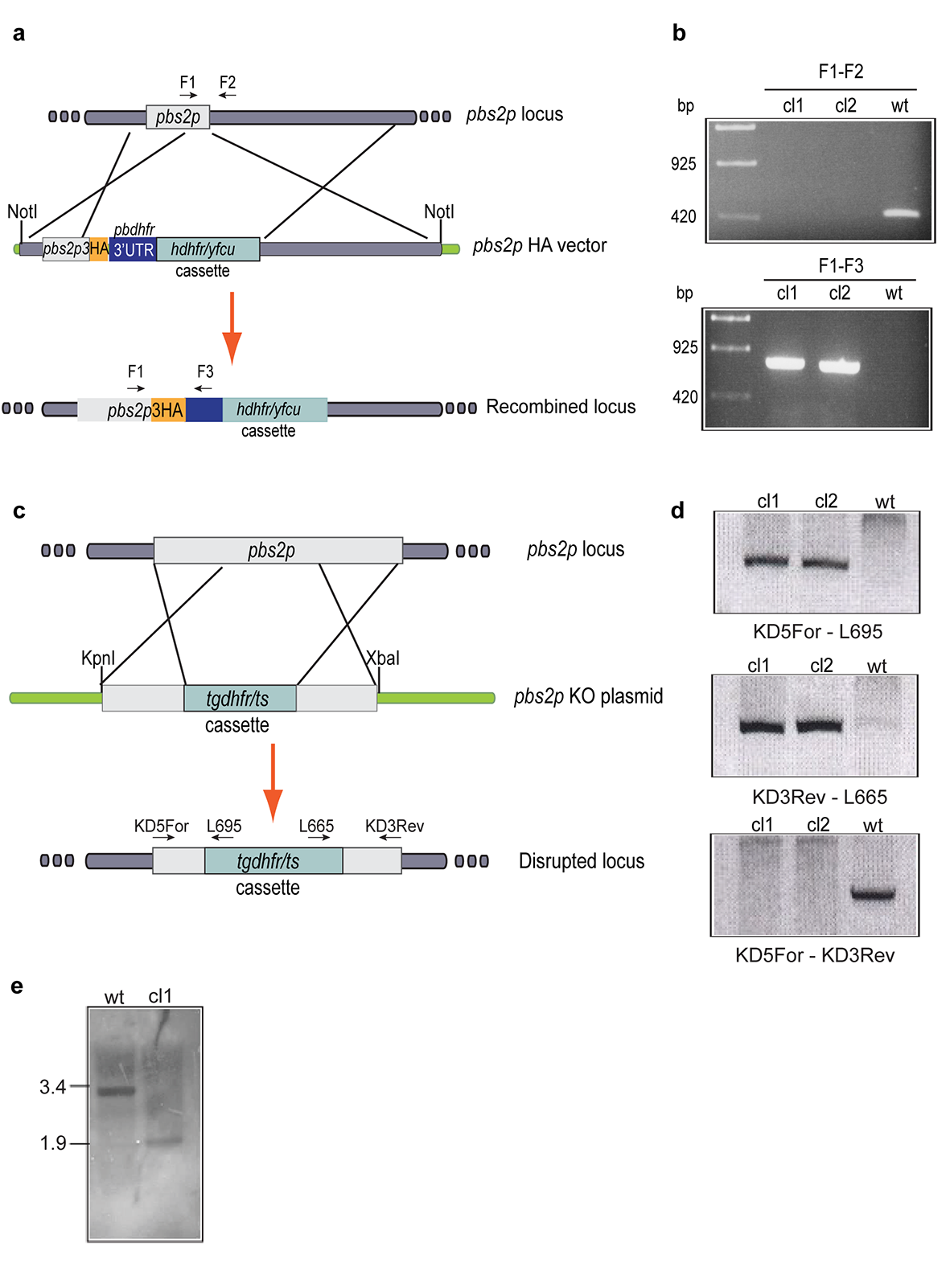

Supplement: S2 Fig — (a) Schematic representation of the gene targeting strategy used for the tagging of pbs2p with a triple HA tag (3HA tag) using PlasmoGEM vector. Upon recombination pbs2p is tagged with a 3HA tag and the endogenous pbs2p 3’ UTR is replaced with the pbdhfr-ts 3’ UTR. Wild type specific (F1-F2) and replace-ment specific primers (F1-F3) are indicated by black arrows. The vector was digested with NotI prior to transfection. (b) PCR genotypic analysis of the derived transgenic clones (cl1 and cl2). The primer pair F1-F2 amplifies the wt locus containing the endogenous pbs2p 3’ UTR. Failure to amplify this 430 bp fragment, indicates absence of the wt locus from genomic DNA isolated from all clones. Primer pair F1-F3 amplifies a fragment corresponding to the replaced locus. (c) Schematic representation of the replacement strategy used to generate s2p(-) parasites. Upon double cross-over recombination, part of the gene is replaced with the selectable marker tgdhfr/ts. Restriction enzymes KpnI and XbaI have been used to linearise the vector. (d) PCR genotyping using primer pairs KD5For-L695 and KD3Rev-L665 for 5’ and 3’ integration respectively and KD5For-KD3Rev to show absence of the wt locus in the obtained clones. (e) Southern blot analysis of genomic DNA isolated from WT and s2p(-) parasites, using a biotin labelled probe for pbs2p. The probe hybridizes to a 3.3 kb fragment in WT and a 1.9 kb fragment in s2p(-) parasites. (TIF) [file pone.0170260.s002.tif]
