# Supplementary material for: Targeted Deletion of a Plasmodium Site-2 Protease Impairs Life Cycle Progression in the Mammalian Host
Source: PLoS One. 2017 Jan 20;12(1):e0170260. doi: 10.1371/journal.pone.0170260 (PMC5249076; doi:10.1371/journal.pone.0170260)
Supplement: S1 Table — (PDF) [file pone.0170260.s003.pdf]

Supplementary Table S1: Primers used in this study

Experiment	Name	Sequence (5´→ 3´)
Genotyping	KD5For	GGTACCATGACTTCAAGAAATATAATATCA
	KD5Rev	AAGCTTATATAAATTTCCTGGTATGC
	KD3For	GATATCTTACAACTGCATTTTATAAGA
	KD3Rev	GGATCCTCACATTTTTTTGAGATATGAA
	L695	ATGTCCATACAACTATATCCGAAC
	L665	GTTGAAAAATTAAAAAAAAAC
	F1	CGAGCCATATTTGCCTTATTTC
	F2	GGATATATATGCATATAATAATGC
	F3	GATTAGCATAGTTAAATAAAAAAAGTTG
Expression data	qS2P-F	CTTGGATTTGATGGATGGGG
	qS2P-R	AAGAAGTGGACACACGTAAGA
	qGFP-F	GATGGAAGCGTTCAACTAGCAGACC
	qGFP-R	GCTGTTACAAACTCAAGAAGGACC
	qPbHsp70-F	TGCAGCAGATAATCAAACTC
	qPbHsp70-R	ACTTCAATTTGTGGAACACC
	qmGAPDH-F	TGAGGCCGGTGCTGAGTATGTCG
	qmGAPDH-R	CCACAGTCTTCTGGGTGGCAGTG
	Pb18S-F	AAGCATTAAATAAAGCGAATACATCCTTAC
	Pb18S-R	GGAGATTGGTTTTGACGTTTATGTG
